# The Role of Antenatal Ultrasound Scans in the Early Detection of Alobar Holoprosencephaly: A Case Report

**DOI:** 10.7759/cureus.70843

**Published:** 2024-10-04

**Authors:** Mariam E Mohamed, Shymaa R Ahmed, Elsayed M Elsayed Ahmed, Eman H Ibrahim

**Affiliations:** 1 Clinical Sciences, College of Medicine, Gulf Medical University, Ajman, ARE; 2 Radiology, Thumbay Hospital, Fujairah, ARE; 3 Pediatrics, Kalba Hospital, Fujairah, ARE; 4 Pathology, Al-Azhar University, Cairo, EGY; 5 Pathology, Gulf Medical University, Ajman, ARE

**Keywords:** alobar holoprosencephaly, anomaly, antenatal, atrioventricular defect, clubfoot, omphalocele, ultrasonography

## Abstract

Holoprosencephaly (HPE) is a developmental defect that affects the brain structure due to failure or incomplete division of the prosencephalon at the third week to the fourth week of gestation into cerebral hemispheres. Although the exact cause of HPE remains unclear, it is suspected to result from a combination of genetic and environmental factors. We report a case of alobar HPE. This case was diagnosed on antenatal ultrasonography in a 42-year-old female (Gravida 3, Para 2) with an unremarkable family history and absence of any risk factors. The association of alobar HPE with an atrioventricular canal defect, left-sided clubfoot, and omphalocele, in this case, constitutes a rare fetal morphological presentation. This case highlights the crucial role of regular antenatal ultrasound scans in the early detection of fatal anomalies like alobar HPE.

## Introduction

Holoprosencephaly (HPE) is a developmental defect of the embryonic forebrain (prosencephalon) that can result from a monogenic cause or occur as part of a genetic syndrome (e.g., trisomy 13 or 18). It is commonly associated with central nervous system (CNS) defects and midfacial abnormalities, which correlate with the severity of the CNS defects as described by William DeMyer in 1964 as “the face predicts the brain” [1]. CNS defects may include incomplete separation of the cerebral hemispheres, absence of the corpus callosum, presence of a single ventricle, or fused basal ganglia. Midfacial defects can manifest as hypotelorism, cyclopia, proboscis, or cleft lip. Additionally, patients with HPE may experience intellectual disability, movement disorders, epilepsy, and endocrine system involvement such as pituitary dysfunction [2]. HPE is classified into four main types based on severity: alobar, semilobar, lobar, and the interhemispheric variant (MIHF). This developmental defect is relatively rare, with a prevalence of 1.31 per 10,000 live births and stillbirths according to a multicenter study [3,4]. Various imaging techniques are used to diagnose HPE depending on the severity of the condition. Prenatal ultrasound (US) is effective in detecting facial abnormalities associated with severe HPE, particularly the alobar subtype, as early as the first trimester. Fetal magnetic resonance imaging (MRI) provides a more detailed assessment of subtle malformations identified in prenatal US, especially in the third trimester, and assists in guiding appropriate referrals and recommendations [5].

## Case presentation

**Figure 1:**
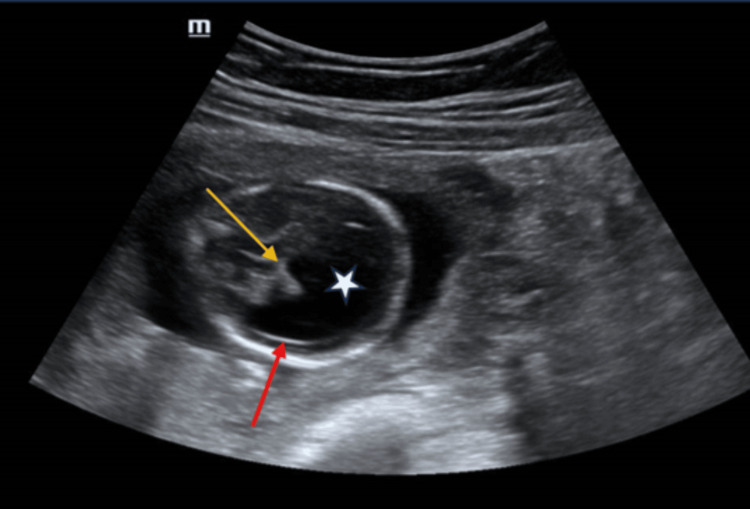
Axial ultrasound image of the fetal brain shows a large central monoventricle (white star) with a surrounding small rim of cerebral cortex (red arrow) and fused thalami (yellow arrow).

**Figure 2:**
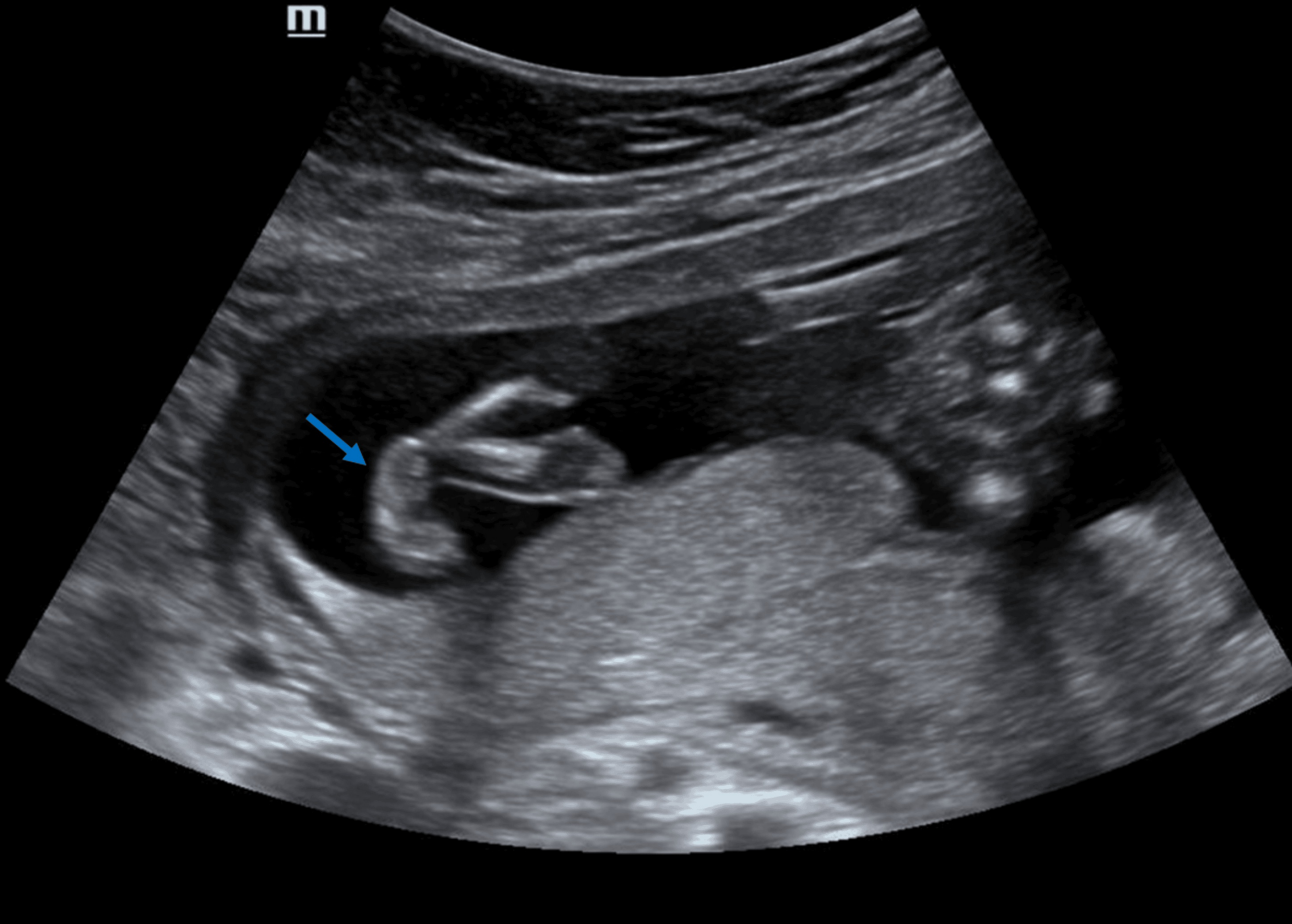
Ultrasound image of the fetal limb shows clubfoot (arrow).

**Figure 3:**
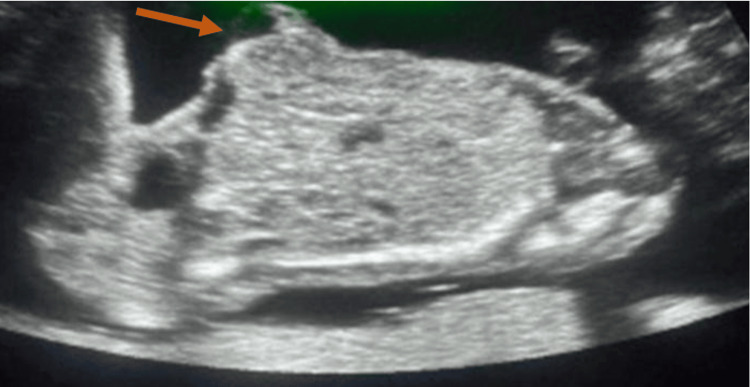
Ultrasound image of the fetal abdomen shows multiple hyperechogenic loops herniating into a membrane covering defect at the level of the umbilicus indicating omphalocele (arrow).

**Figure 4:**
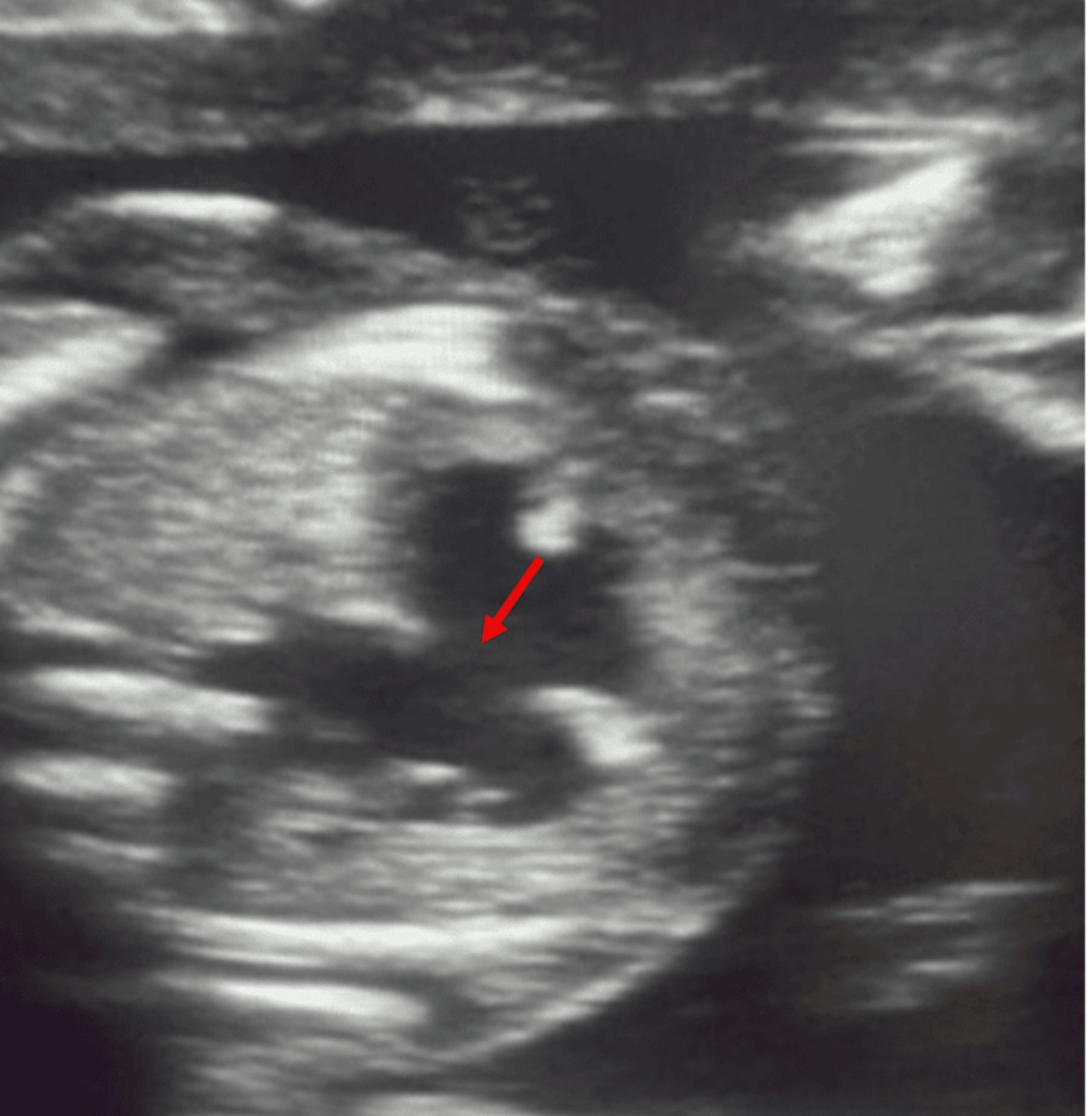
The fetal heart shows the AV canal defect (arrow). AV: Atrioventricular

A 42-year-old pregnant woman (Gravida 3, Para 2) presented to the radiology department for an antenatal check-up at 16 weeks of gestation. Her first two children were boys, and both were healthy. She was a non-smoker, had no history of diabetes mellitus or teratogenic diseases, was not exposed to radiation or toxic substances during her pregnancy, and was not on any medications. There was no history of consanguinity in her marriage, and her family history was unremarkable for any congenital malformations. Her general physical examination was normal, and clinical evaluation indicated a gestational age of 15-17 weeks. US revealed a single live intrauterine fetus with a gestational age corresponding to 16 weeks. The fetal skull showed replacement of the supratentorial brain tissue with cerebrospinal fluid, a thin rim of cerebral parenchyma, a central monoventricle, and fused thalami. In addition, the midbrain structures (cavum septum pellucidum, corpus callosum, and third ventricle) were absent (Figure 1). Clubfoot (Figure 2) and omphalocele (Figure 3) were also detected. The infratentorial brain (posterior fossa structures) was normal. US examination of the heart revealed an atrioventricular (AV) canal defect (Figure 4).

**Figure 5:**
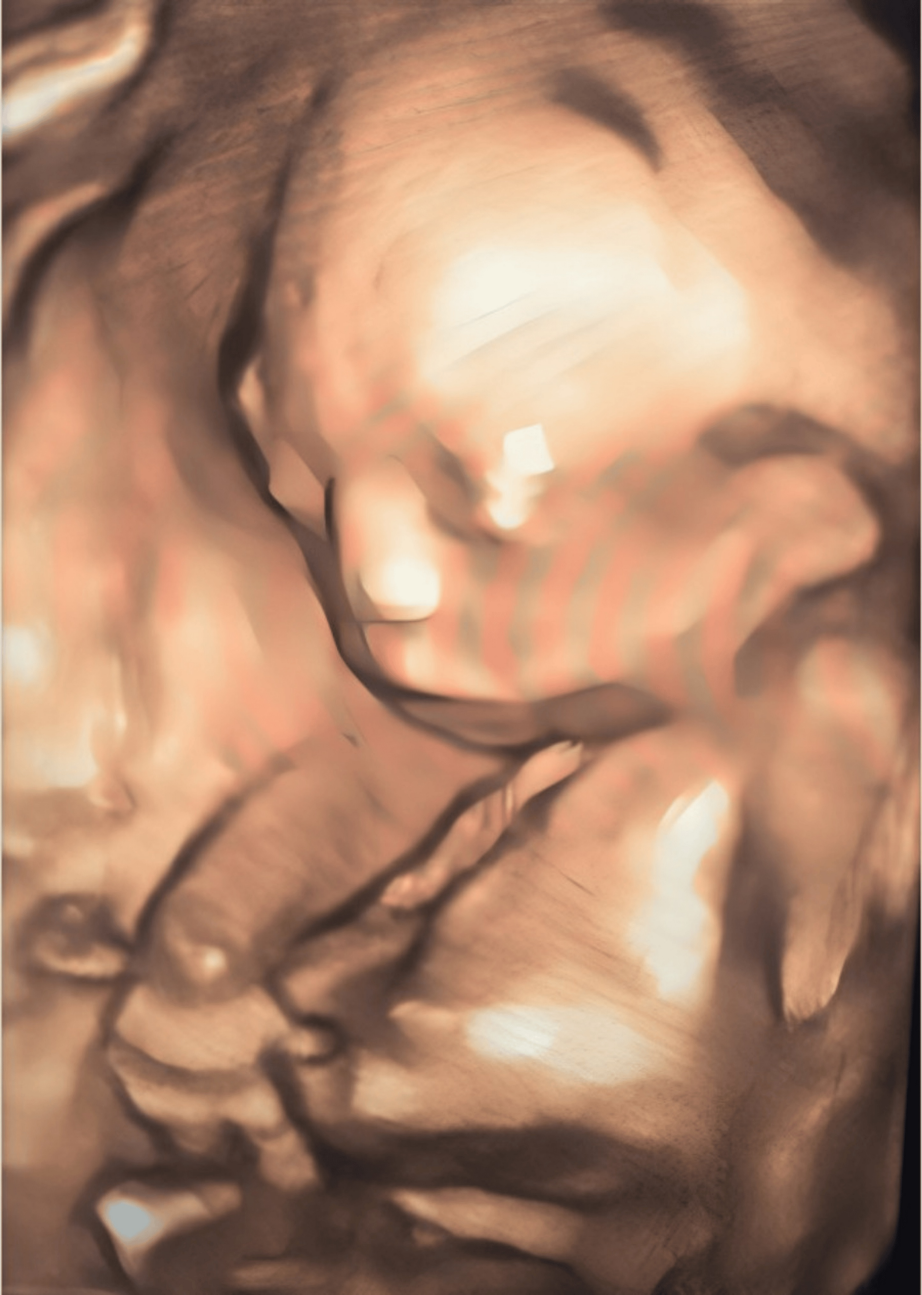
3D ultrasound image reveals the absence of midfacial abnormalities such as cyclopia, proboscis, or cleft lip.

The rest of the examination of the fetus revealed no further abnormalities in other organs. Polyhydramnios was absent. Fetal tone, activity, and movements were normal. No midfacial defects were detected in the three-dimensional (3D) US findings (Figure 5).

## Discussion

HPE is a developmental defect that affects the brain structure due to failure or incomplete division of the prosencephalon at the third week to the fourth week of gestation into cerebral hemispheres. During normal development, the disk-like neural plate folds inward to create a neural tube in the third embryonic week. Neural crest cells then arrange along the neural tube and migrate to specific locations. After the neural tube fuses at the midpoint, it continues to close both rostrally and caudally, completing the closure of the rostral and caudal neuropores [6]. The neural tube gives rise to the three primary brain vesicles: the prosencephalon, mesencephalon, and rhombencephalon. By the fifth week of intrauterine life, the prosencephalon further divides into the telencephalon and diencephalon. The telencephalon develops into the two cerebral hemispheres, while the diencephalon forms the thalami, hypothalamus, and basal ganglia [7]. Disruptions in embryologic growth and development at this stage can lead to the development of HPE. Although the exact cause of HPE remains unclear, it is suspected to result from a combination of genetic and environmental factors. Several genes have been associated with HPE, including zinc finger protein of the cerebellum 2 (ZIC2), Sonic hedgehog, sine oculis homeobox, homolog 3 (SIX3), and transforming growth factor beta-induced factor. HPE can be associated with chromosomal abnormalities, particularly trisomy 13. Other chromosomal abnormalities include triploidy and trisomy 18, with trisomy 18 being much rarer [6,8]. Some environmental risk factors such as retinoic acid [6,9], maternal diabetes mellitus, smoking [6,10], and substance abuse during early pregnancy [6,11] are linked to HPE. The diagnosis of HPE can be made early in the prenatal period using US and MRI imaging modalities. In severe cases, such as alobar HPE, US imaging has an excellent sensitivity and specificity to detect the anomaly as early as the first trimester. Early prenatal diagnosis of alobar HPE allows the mother to consider a safe termination of pregnancy before 20 weeks of gestation [12]. In our case, the antenatal US scan successfully detected the key findings of alobar HPE at 16 weeks of gestation. These findings included a fused central monoventricle with a thin rim of the cerebral cortex, fused thalami, and the absence of midline structures such as the septum pellucidum, corpus callosum, and third ventricle. There were no detectable facial defects in the 3D US. However, other anomalies, including clubfoot, an AV canal defect, and omphalocele, a rare visceral defect associated with alobar HPE, were identified. These findings might suggest a syndrome commonly linked with HPE, such as trisomy 13 or trisomy 18 [13]. Nevertheless, this cannot be confirmed, as no genetic testing or karyotyping was performed due to the mother's decision to proceed with medical termination of the pregnancy after being counseled about this option. The prognosis for HPE is generally poor and varies depending on the type and severity of the brain abnormalities and the extent of facial defects. Alobar HPE is the most severe form and is incompatible with life, often necessitating medical termination of the pregnancy if diagnosed early through antenatal scans [14,15]. Even when babies with alobar HPE are born, their life expectancy is extremely low; only 50% survive beyond 4 to 5 months, and of those, only 20% reach 12 months of age [16,17].

## Conclusions

Alobar HPE is the most severe form of HPE, and regular antenatal US scans play a crucial role in the early detection of this fatal anomaly. Early detection facilitates the legal medical termination of the pregnancy, helping to avoid psychological and emotional trauma for the parents. This case is presented due to its rare occurrence and the unusual findings associated with it, highlighting the importance of US imaging in the early detection of such abnormalities.
